# Ethics in Design and Implementation of Technologies for Workplace Health Promotion: A Call for Discussion

**DOI:** 10.3389/fdgth.2021.644539

**Published:** 2021-08-20

**Authors:** Charlotte Christina Roossien, Marlon de Jong, Anne Maria Bonvanie, Els Lisette Maria Maeckelberghe

**Affiliations:** ^1^Department of Rehabilitation Medicine, University Medical Center Groningen, University of Groningen, Groningen, Netherlands; ^2^Department of Experimental Psychology, Faculty of Behavioural and Social Sciences, University of Groningen, Groningen, Netherlands; ^3^Department of Operations, Faculty of Economics and Business, University of Groningen, Groningen, Netherlands; ^4^Wenckebach Institute for Medical Education and Training, University Medical Center Groningen, Groningen, Netherlands

**Keywords:** privacy, autonomy, generalisation, responsibility, ethics, responsible research and innovation

## Abstract

**Aim:** This study aims to initiate discussion on the ethical issues surrounding the development and implementation of technologies for workplace health promotion. We believe this is a neglected topic and such a complex field of study that we cannot come up with solutions easily or quickly. Therefore, this study is the starting point of a discussion about the ethics of and the need for policies around technologies for workplace health promotion.

**Method:** Based on a literature review, the present study outlines current knowledge of ethical issues in research, development, and implementation of technologies in the workplace. Specifically, the focus is on two ethical issues that play an important role in the worker–employer relation: privacy and autonomy.

**Application:** Two cases indicative for a multidisciplinary project aimed at developing and evaluating sensor and intervention technologies that contribute to keeping ageing workers healthy and effectively employable are explored. A context-specific approach of ethics is used to investigate ethical issues during the development and implementation of sensor and intervention technologies. It is a holistic approach toward the diverse field of participants and stakeholders, and the diversity in perceptions of relevant values, depending on their respective professional languages.

**Discussion:** The results show how protecting the privacy and autonomy of workers cannot be seen as stand-alone issues, but, rather, there is interplay between these values, the work context, and the responsibilities of workers and employers. Consequently, technologies in this research project are designed to improve worker conscientious autonomy, while concurrently creating balance between privacy and health, and assigning responsibilities to appropriate stakeholders.

**Conclusion:** Focusing on a contextual conceptualisation of the ethical principles in the design and implementation of digital health technologies helps to avoid compartmentalization, out-of-context generalisation, and neglect of identifying responsibilities. Although it is a long reiterative process in which all stakeholders need to be included in order to assess all ethical issues sufficiently, this process is crucial to achieving the intended goal of a technology. Having laid out the landscape and problems of ethics around technologies for workplace health promotion, we believe policies and standards, and a very overdue discussion about these, are needed.

## Introduction

A major challenge caused by the ageing workforce is to keep workers fit for work (1) to achieve a sustainable workforce. Technological interventions can assist to maintain individual workability, for instance, by addressing the needs of ageing workers in an objective manner (2) and creating balance between individual capacity and workload through well-designed workplace health interventions (1). Examples of digital health technologies that are applied in the workplace are accelerometers, measuring bending, standing, and walking activities (3) and wearable sensors for measuring fatigue (4). Technologies such as these are aimed at automatically measuring and intervening worker behaviour by giving (automated) feedback through digital means such as smart phones or stand-alone digital applications. These digital health technologies are used in addition to existing workplace health practises.

Research into the design and implementation of digital health technologies is surrounded by ethical issues that require responsible research. It is important to think about what impact this technology might have on individuals who are targeted as potential users or even on society as a whole. Responsible research and innovation (RRI) is a field of science that aims to highlight these socio-ethical issues in research and innovation practises (5, 6). In the past decade, new knowledge and guidelines have been developed that empower researchers to incorporate the responsibility of the researcher throughout the innovation process (7, 8), focussing on anticipation of (un)foreseen ethical qualms, reflexivity on one's own role, inclusion of diverse perspectives, and responsiveness to societal needs. Studies that describe the employed techniques to overcome the socio-ethical issues in development are lacking (9), and publications in the field of responsible research and innovation still struggle with three critical problems: compartmentalization, generalisation, and vagueness about responsible use (10–13).

Compartmentalisation of focus in the current setting refers to the focus on one part of the development or implementation phase, while not including the tension between the intended and actual use of a technology. Until now, studies have mostly focused on ethical issues in either the design of new technologies (4, 10, 14, 15) or ethical issues in the implementation of existing technologies (11, 12, 16). When considering the issues surrounding implementation, technologies are usually taken as a given and the inherent values in the design are not questioned. This situation does not do justice to reality: if design and implementation do not acknowledge ethical concerns and intended values of each other, the final use of the technology will not reflect the intentions of both sides. A broader view on the transition between design and implementation is called for (17) to facilitate responsiveness between these phases of RRI.

An example of compartmentalization can be found in the field of health care innovation. New innovations are often developed from the viewpoint of a technology-enthusiast designer, whereas many nurses and caretakers are not digitally skilled (18). The ethical concerns of designers might be solved by a technical solution; however, due to lack of technical skill, the users do not use the technology properly and bypass these ethical concerns. Take, for instance, the use of smart glasses in health care. The smart-glass is used to share images of patients in a healthcare institution with colleagues in order to get a second opinion. This is a privacy issue. Therefore, the design forces people to first agree to the terms, and then call the colleague, using the tiny screen on the smart glass. This action, however, is difficult and requires training and practise. For digital starters, this is an insurmountable problem. Instead, they use the glass by letting a colleague set it up before they enter the room (thereby violating the right to privacy of the client) or by using other applications to facilitate the sharing of images, such as WhatsApp video calls. This makes the ethical issues and risks of privacy violations even bigger.

In the case of the second problem, generalisation, a single issue is identified as a core problem and addressed in a general way without attention to the specific context. For example, privacy is one of the significant issues in the development and application of new technologies that collect large amounts of data of individuals (19–22). However, most analyses of privacy issues focus on technologies that are used in the public space. These analyses do not necessarily fit other important contexts, such as use of sensor technologies in the work environment designed for health promotion. With regard to new technologies designed for the work environment, specific issues that concern privacy in the worker–employer relationship remain unaddressed. Additionally, discussion lacks about how privacy is embedded in the broader context. For example, specific features in the design of digital health technologies intended to protect the privacy of the user can actually decrease the autonomy of the user. This could be specifically problematic in the work environment. That is, research suggests that workers experience (12) and fear (23) a loss of privacy and autonomy due to the use of technologies and (preventive) health interventions in the workplace. This lack of context-specific knowledge of both privacy and autonomy results in ethical issues that are not appropriately addressed in the development of new technologies.

Albeit not an example from the workplace, the recent development of apps to prevent the spread of COVID-19 has illustrated this problem of generalisation fairly well. During the development of these apps, one single issue, i.e., privacy, was identified as the core problem, while other ethical issues were not addressed as much as they ought to have been (24). Based on 349 interviews with participants from nine European countries, Lucivero et al. (25) showed that, instead of or besides fear of privacy violations, people were hesitant to use COVID-19 applications due to other issues, such as scepticism of feasibility and fear of reduced autonomy. In most European countries, the application was eventually used by only a small part of the population, which not only vastly reduced its effectiveness, but, potentially, also reduced trust in and potential use of future applications with similar goals (24). This mismatch between values addressed by the developers and the values that are important to the user shows that generalisation is a common problem that is not addressed properly in the design of technologies. Even though, as this example illustrates, generalisation has the potential to have a large impact on the outcomes and use of a technology.

Finally, the topic of responsible use of digital health technologies remains vague and insufficiently addressed. Providing transparency about responsible use, as well as identifying who is responsible, is lacking. For example, Leclercq-Vandelannoitte (12, p. 151) observed that, in the use of ubiquitous technologies in the workplace, neither workers nor employers recognise who is responsible for technology, nor do they understand the importance of responsible use of these technologies. Furthermore, designers do not provide insight into the responsible use of their designs. Thus, identifying responsible use is notoriously difficult due to interdependent design-use dynamics (26). These dynamics entail that design and use continuously impact each other because a particular function is often the reason for the design of a technology. However, the adoption of the design can substantially change the function. An example is the innovation of the short message service (SMS), which was designed to enable mobile owners to receive messages about incoming voicemail as well as bills from their service provider (27). However, SMS developed into a primary function for communication between individuals, thereby posing additional design demands as well as responsibilities that were not relevant to the original function.

Although, in principle, new sensor technologies are developed to support the user, they can have unforeseen consequences that are unintentionally harmful to the user or to society (28). For instance, health-insurance companies in the Netherlands ask their customers to share their personal activity data, monitored *via* a pedometer or step counter on their phones. By doing so, these individuals could earn back part of their insurance fees. Although these marketing strategies are being framed in a way that they are beneficial to the user, there are other values at stake (e.g., inequality between individuals with different socioeconomic status and use of health data by the insurance company). While activity trackers were initially developed in order to help individuals self-manage their health, commercial organisations now make use these simple devices for their own commercial benefits.

Both the example of the SMS and the activity tracker show that the interdependent design-use dynamics of such a technology makes it difficult to predict how it will be used in the future and whether or not it will be used as intended. However, this difficulty should not hinder designers from at least outlining the responsibilities inherent in their designs.

This study aims to overcome these issues of generalisation and compartmentalization and additionally identify relevant responsibilities in the design and implementation of digital health technologies in the workplace. We want to initiate a discussion about the ethical issues surrounding workplace health promotion and the role of technologies. We believe this is such a neglected field that we cannot come up with solutions easily or quickly. Therefore, the present study is an invitation to engage in a discussion about the problems we encountered. Ideally, work health considerations and responsibilities of employers would be set in a trajectory of health over the lifetime of work. In this paper, the focus will be limited to the problems of developing and introducing technologies. These technologies, however, are intended to have an effect on health over the lifetime of work. We also want to point out that the problems we signal are not new but are acerbated by the introduction of currently available technologies. The examples we use might seem quite simple, conventional, and not new at all. However, they show how slow we are to come up with solutions and how far behind we are in the discussion about ethical considerations on technologies in the workplace.

First, the present study outlines current knowledge of ethical (and legal) issues on the implementation of technologies in the workplace, specifically focusing on the two ethical issues that play an important role in the worker–employer relation: privacy (29) and autonomy (12, 23). Secondly, two cases were explored, using a context-specific approach of ethics to investigate these ethical issues during the development and implementation of sensor and intervention technologies for health purposes in the workplace. This context-specific approach arose from the diversity of participants and stakeholders and differences in languages (different academic disciplines; fields of application) used.

### Privacy of Workers

Employers are obligated to guarantee a safe working environment for their workers and should be reluctant to meddle with the private lives and personal data of the workers. Interfering with health behaviour of workers, especially as connected to lifestyle, is dubious at best. It targets individuals (at work and in a personal setting) instead of organisational and collective problems, even if the goal is sustainable employability (30). Therefore, sensor and intervention technologies should comply with several criteria to ensure worker privacy.

Firstly, according to the EU General Data Protection Regulation, Article 15, section Introduction (31), the worker should be able to access all personal data and outcomes of sensor and intervention technologies without the interference of others. Secondly, the employer should not have access to data and outcomes of individual workers or be able to derive these outcomes from group data (30, section Conclusion: Call for an Overdue Discussion). Current regulations on data collection and individual privacy limit the possibilities of data sharing (31). As stated in Article 6, section Introduction, Subsection d of the GDPR, data processing is only valid if it is necessary to protect the vital interests of the subject, hence, a life-or-death situation.

Legally, data sharing at a group level is only allowed if the data do not contain identifiable information, such as personal data traceable to individuals (30, section Case Study 2). Specifically, when it comes to sensor data that cross the border between work and private life, serious legal concerns arise regarding data and health privacy (32). It could be argued, however, that sharing digital health data with relevant actors, such as health and safety workers, is beneficial for workers in specific contexts. In case of workplace improvements, the use of personal data could help to improve working conditions. The GDPR, however, does not provide a legal basis for the exchange of personal data in these specific relationships (33), making it difficult to use digital health data in the work environment, even if it can improve health of a worker.

A needs assessment among workers with physically demanding work identified a demand for sensor and intervention technologies (29). However, respondents expressed concerns about what would happen with the personal data retrieved by the sensors, fearing their privacy would be violated, especially if employers had access to the data. These apprehensions confirm the findings of other studies (34, 35). The GDPR, as described above, offers an extensive legal framework protecting the rights and freedoms of data subjects, ensuring data minimisation, informed consent, good practise *via* the data protection impact assessment (DPIA), and privacy by design (31, 36, 37). Although this legal framework is intended to protect workers, in some cases, workers are not necessarily protected by it, nor do they want to be protected in this manner. That is, workers also declared that they would share their data with their employers to explore possibilities to improve working conditions if they could retain full ownership of the data (29).

Absolutizing a legal framework potentially leads to narrowing the fundamental questions of why privacy is an essential moral value. Data protection is significant to ensure privacy, but it does not embrace a comprehensive understanding of the concept. Numerous scholars have warned against a reductionist conceptualisation of privacy as merely about the protection of the personal sphere, raising questions about possible conditions under which this protection can be overruled (21, 37–41). They have argued for a broader understanding of privacy based on a reflection of practise and context. A legal framework for privacy by nature is fixed; however, privacy as a value should be shaped by each situation. Nissenbaum (21, p. 2) succinctly summarised this concept: “*What people care about is not simply restricting the flow of information but ensuring it flows appropriately*.”

Privacy as an essentially contested and malleable concept is dependent upon, amongst other things, the context in which it is examined, and the social and technological circumstances that apply to this context. As the theoretical debate about privacy continues, there is a need for a context-specific approach. Mulligan et al. [(37), p. 15] have suggested an approach based on four questions: “*While dilemmas between privacy and publicity, or privacy and surveillance, or privacy and security persist, the question we more often face today concerns the plurality available to us amidst contests over privacy: Which privacy? For what purpose? With what reason? As exemplified by what?*”. These questions enable researchers and practitioners to pragmatically define the relevant characteristics of the applicable notion of privacy.

### Worker Autonomy

A significant challenge for a workforce that will continue working into older age is to keep workers fit for work (1). Van der Klink et al. [(42), p. 74] suggest to focus on sustainable employability based on a capabilities approach. Maintaining and supporting the ability of workers to continue working depend on the adaptation of work behaviour to changing circumstances. Worker autonomy in the self-regulation of work behaviour is crucial in this process (43). Hence, organisations are introducing an increasing number of digital health devices on the work floor with which workers can regulate their tasks and work behaviour to ensure the autonomy needed for self-regulation.

Technological interventions can assist in maintaining ability of workers to work, for instance, by developing technology that addresses the needs of ageing workers objectively, such as interventions that increase physical activity and ergonomically flexible workplaces (2). Thus, digital workplace health interventions can create a balance between capacity and workload of workers (1), and sensor technologies, such as activity monitors and heart rate monitors, can accurately monitor a workload. Additional intervention technologies, such as smart chairs (44, 45), can support workers in altering behaviour to prevent and solve health problems effectively.

Workers are willing to adopt sensor technologies that are perceived as useful (34, 35), but willingness of workers to use these technologies depends on the addressing of concerns about data security and technology misuse (35). Philosophically, autonomy is complex, and caution is necessary to narrow the notion of autonomy to an idea of self-determination. Autonomy is a normative idea that directs actions governed by a responsible commitment to the norms with which one binds oneself. It can be about willed ideals of one as well as a commitment to the norms and standards people encounter and adopt because of a specific setting, such as the workplace. Thus, autonomy, also referred to as ‘conscientious autonomy’, (46) covers the high moral values that direct lives of peoples as well as small practical commitments that shape ordinary happenings. For instance, if someone values being healthy, practical commitments could include walking to work instead of driving and taking the stairs instead of riding in an elevator.

### Responsibility in the Work Environment

The ultimate responsibility for safeguarding the work environment lies with employers. Employers are responsible for the capabilities of their workers, actively preventing harm and accidents (13, 47). For workers who labour physically, employers must protect safety of workers *via* periodic occupational health examinations and safety monitoring (47). Despite limited access of employers to the outcomes of regular health checks, this examination protects workers because occupational physicians can access health data and warn workers of potential issues while bound to professional confidentiality.

To protect workers while using sensor and intervention technology, all stakeholders must be responsible for the proper use of these technologies (48), although employers may have different views on this responsibility than workers (30). Both workers and employers acknowledge the responsibility to prevent harm in the workplace. However, many employers consider the responsibility to stay healthy and fit for the job to be the responsibility of the worker, while workers embrace autonomy in their lifestyle choices (30). These contrary views see health as either a safety discourse or a lifestyle discourse (49). Nevertheless, the responsibilities of workers and employers in both discourses must be examined through context-specific ethics to prevent ambivalence in the worker-employer relationship (30).

## Practical Examples

### Project Description

The project SPRINT@Work is an EU-funded interdisciplinary project aimed at developing and evaluating sensor and intervention technologies that contribute to keeping ageing workers healthy and effectively employable (45, 50–56). These health-related technologies were developed and implemented by researchers and engineers from a variety of disciplines (cognitive neuroscience, information management, biomedical engineering and rehabilitation medicine, community and occupational medicine), in collaboration with companies. The developed sensor and intervention technologies lead toward an automated, digital process of behavioural assessment of employees for health self-management purposes. Cognitive neuroscience and information management were represented by one professor and one Ph.D. candidate, biomedical engineering and rehabilitation medicine were represented by two professors and one Ph.D. candidate, and community and occupational medicines were represented by two professors, one postdoctoral researcher, and one Ph.D. candidate. The four Ph.D. candidates acted as executing researchers.

### Procedure: Context-Specific Approach of Ethics

In several intervision sessions between the executing researchers and, later, the entire project team, the following issues were addressed: (a) whether the legal framework of privacy identifies sufficiently what is at stake in the context of the development and implementation of sensor technologies for sustainable employability, and (b) whether self-management devices aimed to promote self-regulation can assist in enabling the autonomy of workers. The team developed a conceptual framework that contextualises data protection and privacy issues as well as the notion of worker autonomy. This framework of context-specific ethics was helpful in both designing and implementing sensor technologies, and it functioned as a benchmark for the researchers. That is, during the project, the researchers continuously checked whether their proposed design was in line with context-specific ethics. Additionally, this normative framework was continuously adapted, using insights from the executed studies.

**Figure 1:**
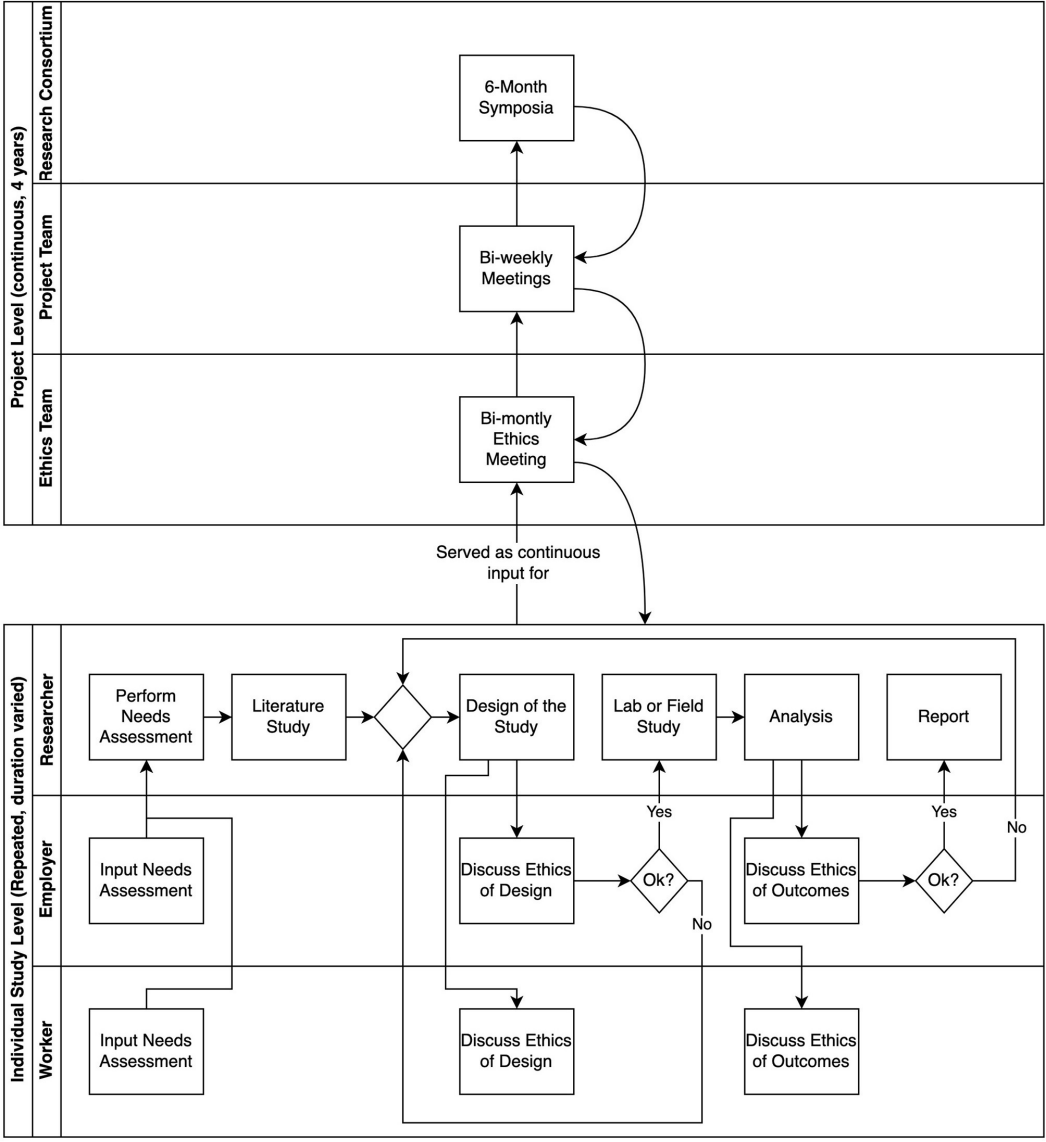
Research flow SPRINT@Work.

Figure 1 shows how the research process during the project SPRINT@Work took place. The researchers involved in SPRINT@Work executed studies individually, while discussing ethical issues with the employers and workers that participated in their studies. The researchers continuously interacted with fellow executing researchers and an ethicist in the ethics team. This ethics team then shared and discussed findings with the project team, including supervising researchers, and higher-level findings were shared with the consortium. The outcomes of the meetings with the consortium, project team, and ethics team were used to improve the studies of individual researchers.

### Case Studies

The present study highlights two case studies that were performed by the researchers of SPRINT@Work. The first case study was about monitoring the core body temperature as a parameter of heat stress of firefighters. The objective of this study was to validate a wearable noninvasive core thermometer to monitor the core temperature of firefighters during firefighting simulation tasks (54). The second case study was about a research on health self-management applications in the workplace of health care workers. This study aimed at investigating whether use of sensor and intervention technology enhances the autonomy of workers in self-regulating their health-related behaviour (50).

In both studies, the employer decided whether the study could be executed within the company. Thereafter, workers could voluntarily participate in the field studies. The employers were not allowed to oblige the workers to use the sensor technology, nor could they ask for data if the workers voluntarily used a sensor technology (57). The intentions were articulated according to the declaration of Helsinki on research involving human subjects (58), stating that participants should voluntarily give informed consent.

## Case Study 1: The Case of Firefighters

During their job, firefighters are exposed to a high thermal load due to heavy physical activity, external heat exposure from fires, and the wear of highly insulated protective clothing (54). This can lead to heat stress and subsequent related health problems, such as exhaustion, dehydration, mental confusion, and loss of consciousness (59). In more extreme cases, heat stress can cause permanent damage and can even be life-threatening (60, 61), thereby affecting the long-term health of the firefighter, affect productivity, and risk perception, and cause safety problems (59). There are large differences between individual firefighters regarding how their body copes with excessive heat. Therefore, general guidelines for duration of exposure to heat are not sufficient for the whole population of firefighters. To prevent heat stress among firefighters, Roossien et al. (54) aimed to develop a new technology that would allow for monitoring and intervening in real time during potentially harmful work situations.

### Overcoming Compartmentalisation

The firefighting department that participated in the design and development of the intervention indicated a desire for a wearable thermometer to measure the real-time body temperature, because they wanted more insight into heat stress during work. This solution was developed in this case study. The thermometer was worn in-ear and registered the real-time core temperature of the firefighters (54). It is dangerous if the firefighters themselves become distracted by immediate feedback on the obtained data, and they neither have time nor opportunity to monitor the feedback and data from their own sensors. Therefore, it is necessary that other colleagues, such as the captain, monitor the current body temperature of their workers on-site. This way, they are able to intervene when the monitors show changes in the body temperature, which could potentially harm the workers.

During development and testing, the researcher discussed issues regarding data sharing and confidentiality with both workers and captains, as well as with the other researchers, in order to find ways to overcome the potential issues regarding privacy and worker rights (see Figure 1). Legally, an employer cannot ask permission to access the personal data of workers (30, section Case Study 2), even if it is to the advantage and safety of the workers. This issue points to ambiguity in the data protection law on the protection of privacy of workers opposed to the responsibility of the employer to safeguard health and safety of workers. Employers cannot, under any circumstance, use personal sensor data for the protection of health and safety of their workers, even though employers have the responsibility to protect workers from harm in the work environment. An ensuing focus for the research team was to explore how privacy could be conceptualised in the specific context of sensor technologies in the workplace, despite such ambiguity.

### An Agency-Based Approach to Privacy

Following the pragmatic approach of Mulligan, data sharing in the case of the firefighters was analysed to determine what kind of privacy might provide sufficient protection. Control over personal information, such as the core temperature and heart rate of the firefighters, is a critical target for protection. As previously stated, from the perspective of the GDPR, this type of data can only be accessed under stringent circumstances and must be handled by a health professional, who is bound by professional confidentiality. Nevertheless, in the case of a fire, no such health professional is available. Thus, the harm that supposedly would be prevented by enforcing data protection might be superseded by the prevention of more prominent harm. This example illustrates how information becomes ethically and normatively significant, not because it is about specific values such as privacy but because the context allows its use for action. In this case, the possible prevention of overheating. Hence, it is not about what information one has but about what one can do with that information.

Manson and O'Neill (62) called the above explanation an agency-based model of informing and communicating, where it is necessary to analyse what the agent, in this case, the firefighter captain, can do with the private information obtained. If overheating can be prevented, firefighters might want the option to share sensor information with their captain, although the captain is not bound by confidentiality as a health professional. Hence, the permission of the firefighters for the captain to access this information is based on the specific agency of the captain to protect the firefighters from overheating. A different way to protect the privacy of firefighters is making sure firefighter captains are bound by the confidentiality of their own profession.

The answers to questions of Mulligan et al. (37)—“Which privacy? For what purpose? With what reason? As exemplified by what?”—are that, in the case of the firefighters, the privacy at stake is the ownership of personal data obtained by sensor technologies. The purpose of privacy is to give the firefighters control over their data, not only to prevent the employer to use this personal information but also to allow the firefighters to share the data as they deem acceptable. The agency-based model exemplifies this purpose: In an ideal situation, the firefighter can opt to share data for protection from health hazards with the captain, who can act to prevent health hazards but cannot use the data for any other purposes, because the data is formatted in such a way that only the direct hazard of overheating is shown. This could, for instance, be done by using a traffic light figure that only shows whether a situation is safe (green), or a reason to be alert (orange) or immediately withdraw the firefighter (red). In cases where direct indication of this risk of overheating is considered too much of a privacy violation, the agency-based approach could also allow including other health and safety indicators, such as an almost empty oxygen tank or another workplace risk. In this way, an orange or red warning light does not solely give the captain information on health of a worker but also on health and safety risks, in general. This example shows that a narrow interpretation of privacy might result in diminishing safety: If privacy is unidimensional, and the only choice would be to decide to share the data with the employer, either the firefighter would accept more significant risks during the execution of the job because the data would be hidden (as in the GDPR), or the employer would have full access to all data, which could lead to misuse for other purposes.

The case of the firefighters showed a disbalance between what is actually beneficial for the health of the firefighters and the regulations that are meant to protect them. This is a major problem when implementing new technologies in the work environment. Given that the law not yet protects the user in fiduciary relationships in certain professions (31), it is important to acknowledge these design-use dynamics in the design phase of a new technology and come up with solutions that could help overcome this gap in the law. Although some researchers already call for changing the law for fiduciary relationships (33), this would be a long and arduous process. Even if the law would change on this matter, it would still be important to define in which situations data sharing is condoned and with whom sharing health data is necessary. Therefore, the agency-based approach asks for a thorough discussion with all stakeholders involved about what type of data is necessary to share with other actors and with whom in order to protect the health of the firefighters (as can be seen in the process described in Figure 1: level individual study). For instance, is it necessary to share raw data? Or would aggregated data suffice? Is it important to collect data for longer periods of time? Or can the data be removed directly after the fire was put out? But also, who has access to the data? And how can it be prevented that other colleagues have access to the data? This can also be an indirect result of the use of a sensor. What happens, for instance, if one firefighter is called back more often than other firefighters? Agreements on these issues should be strictly documented and revised if necessary.

### Responsibilities of Stakeholders

In the case of the firefighters, the employer is serious about the responsibility for the health of the workers. The GDPR, however, prevents the employer from using personal data to protect firefighters from overheating in an emergency. In this case, the workers are at an impasse. Distraction from the task could cause immediate risks to themselves and colleagues; thus, it is impossible to self-monitor their current health parameters. This discrepancy between the desired situation and current regulations is frustrating for the fire department because the captains wish to protect their firefighters, but the GDPR makes it impossible for captains to use data for the goal of protection of workers.

## Case Study 2

Healthcare workers are often subject to irregular working hours due to shift work. These work characteristics can make it more difficult to uphold healthy habits, such as daily exercise and a balanced diet (63). An unhealthy lifestyle for a healthcare worker not only impacts their employability in the long term (64) but also impacts the view of the public on the healthcare institution, because the healthcare workers are assumed to ‘know best’ about the impact of lifestyle choices on long-term health. Both the issues of long-term health and the exemplary function of their work are well-known to healthcare workers, which is why many of them actively try to keep up good behaviour. In this case study, a healthcare institution asked for an intervention that allows employees to self-manage their health, without having to explain themselves to the employer. An activity tracker supports these workers in their health, because it allows them to monitor their daily behaviour despite the irregular hours and workload, and thereby supports these workers in becoming and staying healthy (65).

### Overcoming Compartmentalisation

The healthcare institution where the study took place is eager to improve and tries to incorporate the ideas of workers into their workplace health promotion policies. The activity tracker used is a tracker developed for the consumer market, meaning that the research team did not have any influence on the specifications of the tracker. During implementation, however, the researchers decided to use proxy user accounts for all users, thereby enabling the researchers to tailor and alter the information that was given to the workers. These adjustments to the messages were intended to limit the impact on worker autonomy (see iterative process Figure 1: individual project—researcher). Apart from the researchers and the participants, nobody had access to the data.

The use of sensor technologies to assist in sustainable employability hinges on offering workers objective feedback and interventions that allow them to self-regulate behaviour. Illustrative for the ideal of autonomy was a participant, self-identified as overweight and unfit, who was eager to experiment with an activity tracker. This activity tracker enabled her to receive automated digital feedback on her daily exercise behaviour. This worker was committed to improving her condition:

*I value a healthy lifestyle. I have difficulties keeping up with that for all sorts of reasons, and this is an opportunity for me to get some nonintrusive and time-saving support. I also would like to be an example for the patients who visit here. They need people like me as role models, people who struggle but make an effort to improve their health*.

She referred to her value of personal health. Receiving an activity tracker did not provide autonomy. However, due to the activity tracker, she could autonomously commit to her value of becoming healthy. This value had a different application in her work context, a healthcare organisation, where she wanted to set an example for others. She wanted to show that increasing daily exercise by walking more and taking the stairs is an essential commitment to improving health. Thus, in the work context, the worker wanted to achieve a healthy lifestyle as well as provide the moral value of being an example. She translated the value of her health and her position at work into a daily practical commitment of taking more steps. Thus, the use of this sensor technology helped her to achieve her ideal.

Nevertheless, the commitment of the worker was not only shaped by a momentous decision to accept the activity tracker. Her commitment was confirmed by making some progress in walking more steps. However, it was disaffirmed when a colleague from higher management saw her waiting for the elevator:

*And then they are supporting “the week of taking the stairs” […], but then, when I am standing in front of the elevator, [colleagues] say, “Oh, are you taking the elevator? We are taking the stairs!” That feels terrible—really terrible*.

This encounter made her question whether the entire experiment was about her improvement in health and realising her values, or whether it was ultimately about organisational control and cost reduction.

This example, although an individual experience, illustrates how personal autonomy can easily be threatened in a work environment if personal values are not acknowledged. Giving workers a health device does not merely provide a means for self-regulation, because the technology is embedded in a context that can promote or disavow the responsible commitment to the norms to which one is bound. This realisation calls for reflection on how the introduction of technology can affect autonomy of workers and how the context of the implemented technology influences the perceptions of autonomy of workers.

Worker autonomy as a prerequisite for health self-regulation was empirically investigated in the study of Bonvanie et al. (50). It examined activity trackers that give feedback information on health-related behaviour to workers. The example of activity trackers is of interest because it is used as a technology that enables workers to self-regulate a healthy lifestyle (66, 67). The underlying assumption was that the use of digital health technologies provides workers with autonomy *via* feedback and the freedom to respond to self-regulate health-related behaviour. Despite adjustments to the messages, intended to limit the impact on worker autonomy, these findings revealed that the use of a sensor technology did not significantly increase perceived autonomy and may have even reduced autonomy under certain conditions, especially for less healthy workers (50). Moreover, the workers who had used an activity tracker to monitor their behaviour before they received an employer-provided device experienced the same decrease in autonomy as the workers who used the activity tracker for the first time. This finding suggests that the activity tracker does not limit the autonomy of workers; instead, perceived autonomy may decrease due to the hierarchical relationship between workers and employers.

### A Conscientious Autonomy-Enhancing Approach

The employer of the health-care institute who participated in this study demonstrated a value for healthy workers. That is, the employer already implemented several other activities and regulations, such as promoting a week of taking the stairs, providing a healthy cafeteria and offering a smoke-free property. Although independent researchers conducted the study, the normative standards of the activity tracker were encouraged by the employer. The goal was to walk 10,000 steps per day and take 10 flights of stairs. Some participants agreed with this goal and internalised the normative standard. Others, however, did not and perceived the feedback as pressure to aim for 10,000 steps. The participants who shared the same value of healthy living as the employer but had other ideas to implement it felt as if the activity tracker forced them to commit to normative standards of someone else.

These findings reflect the idea of conscientious autonomy (46): Autonomy that is committed to willed ideals of one as well as the norms and standards encountered in a particular setting that are adapted as normative. Hence, based on the disbalance between the individual goals and ideals of workers and the norms of their colleagues and employers, one can determine why the autonomy of certain workers declines when using a sensor technology. When implementing technologies or other interventions in the work environment, the employer, therefore, needs to pay specific attention to how the norms and culture in the work environment influence the autonomy of the workers.

Participation in the study and being able to discuss the impact of technologies with different stakeholders within the development process caused the employer to reconsider the current workplace health promotion policies. The employer altered their strategy into a more conscientious autonomy-enhancing approach. This was achieved by including a more diverse group of workers in the decision-making and evaluating the process regarding new technologies and interventions, thereby aiming to facilitate a healthy workplace and a lifestyle for all workers.

### Responsibilities of Stakeholders

Similar to the case of the firefighters, the employer was responsible for the health of the health-care workers. This responsibility of the employer is limited to the work context, while the health of workers is also influenced by their private lives. By providing an activity tracker, the employer is walking a thin line between the work and the private context. One can ask the questions, where does the responsibility of the employer stop? And where does the responsibility of the worker begin? And where do they overlap? Interestingly, the participants in the study of Bonvanie et al. (50) stated that the ability to maintain their health is, partially, the responsibility of the employer, because their work environment has a large impact on this ability, and that their employer took this responsibility quite seriously. Both the employer and the workers experienced the intertwined nature of health, work, and the home environment, and aim to improve the collaboration on improving the overall health of the worker (see process Figure 1: individual level worker-employer).

## Discussion

Previous literature on responsible research and innovation struggled with three major problems: 1) compartmentalisation, 2) generalisation, and 3) vagueness about responsibilities. Rather than developing a theoretical approach to these problems, we highlighted two cases of the project SPRINT@Work. We aimed at describing how we explored the critical ethical issues privacy and autonomy in the development and implementation of digital health technologies in the setting of doing research. A context-specific analysis of both values was employed, keeping previous research and the legal context in mind. For the firefighters case study, this analysis resulted in the description of an agency-based concept of privacy, where it is necessary to analyse and regulate what the agent can do with the private information obtained (62). For the case study of the health-care professionals, this resulted in a conscientious autonomy-enhancing approach to the design and implementation of digital health technologies in the workplace. When this approach is employed, all stakeholders [with a specific emphasis on the user(s)] have to be actively involved in the design and implementation phase in order to achieve the intended goal of the technology, which is to enhance health-related behaviour (46).

### Decompartmentalisation of Focus

Responsibilities for the assessment of risks of the new technology get indistinguishable when a transition between phases occurs (17). More specifically, engineers and researchers might have reflected on the impact of their new technology; however, after the development phase, responsibilities shift toward the user or organisations that implement the technology. They do not necessarily reflect on possible ethical and societal risk, and primarily focus on productivity or increasing product acceptance (12, 68). Ethical concerns arise as soon as technological innovations are introduced (69). Although an ethical script of an innovation shows what the default choices regarding privacy, responsibility, and autonomy are, at the same time, the reaction of the environment to this built-in ethical script plays a significant role. The ethical script is mainly developed by the engineers and researchers who develop the technological intervention, but the response of the user and his/her environment to this ethical script largely determines the privacy of the user and his or her possibility to exercise autonomy. Using a multi-stakeholder approach may help to overcome this problem of compartmentalisation by providing a smooth responsible transition from development to implementation.

In the case studies, the reflection on both design and field experiments involving health-related technologies in the workplace caused both the researchers, employers, and workers to reflect on the interpretation and implications of the concepts of privacy and autonomy (see Figure 1). This approach of integrating development and use of the digital health technology was necessary to successfully implement techniques from the field of RRI, such as reflexivity and responsiveness. The context-specific approach allowed for a cyclic approach, using outcomes from early implementations of technologies as input for further development. As a result, the researchers, employers and workers were able to work together to take unforeseen consequences of the technology into consideration, because they appeared during use by end users. This then allowed the researchers and engineers to alter the technology or the choices that were made during development and implementation.

Both cases show the benefits of including the tension between the intended and actual use in the development and implementation of a new technology. In the case of the firefighters, the balance between safeguarding privacy and safeguarding health could only be reached because the researchers were able to use input from actual use (during job performance). More specifically, due to the interaction between the researchers, workers, and the team captain, the application of the wearable thermometer for use in the workplace could be improved, which consequently benefits the health of the firefighters. In the case of the healthcare workers, the researchers closely monitored the impact of the technology on the autonomy of workers in the workplace. By doing so, they were not only able to reevaluate the benefit of the activity tracker but also caused the employer to reconsider the current workplace health promotion policies and the manner in which these come to be.

### Prevention of Out-of-Context Generalisation

A responsible decision to provide workers with sensor technologies to sustain their employability requires careful analysis of the values at stake in the context of the specific workplace and the individual worker (70). In case of privacy, the GDPR offers a basic framework for the implementation of protection measures, while it also leaves room for interpretation and discussion. The GDPR (3) obligates and ensures that the decisions about data protection taken by the controller, for instance, an engineer or a researcher, are taken with great care, especially when “*processing of the data could result in high risk to the rights and freedoms of natural persons*” [30, section 35 (3)]. In order to help the controller making responsible decisions about privacy of individuals, the data protection impact assessment (DPIA) (71) is developed as a risk assessment method. This includes a multiple stakeholder approach to identify privacy risks. During meetings with stakeholders, a context-specific method of privacy by design is applied to design protection measures that are appropriate for a specific context.

The main focus of DPIA (and of the GDPR) is to protect the privacy of the user without paying much attention to other ethical issues in its analysis. Although it is a step in the right direction, in the development of new digital health technologies, other values, such as health, autonomy and responsibility, and the interplay between these values need to be reflected upon as well. The current study, therefore, used a context-specific approach of ethics (instead of privacy) to assess privacy and autonomy concerns in the workplace.

For both cases, the context-specific approach of ethics helped to identify the best approach to provide a framework of what is at stake in their specific context. Although from a different perspective, both the agency-based model of privacy (62) and the conscientious autonomy-enhancing design (46) can help identify bottlenecks, implicit norms, and courses of action during the development and implementation of new technologies and policies. These two approaches are a source of moral knowledge, given that the experiences in the field informed the researchers about what users value, and the dynamics between the researcher, employer, and user was explored by testing the conceptualisation of ethical principles in the work environment and further adjusted as deemed necessary.

### Making Implied Responsibilities Explicit

Acting responsibly regarding health in the workplace is considered important (30), but employers experience difficulties taking their responsibility, and, in the case of health-promoting technologies in the workplace, other stakeholders find it difficult to share this responsibility. Leclercq-Vandelannoitte (12), in a study about the use of ubiquitous technologies in the workplace, observed that “*despite their prevalence and the importance of their consequences for workers, neither salespeople nor managers seem to be aware of them, feel responsible for them, or appear able or willing to identify the responsibilities involved in this process*.” In the case of workplace health-promoting technologies, responsibility involves multiple stakeholders with a prominent role for the employers (13), engineers (72), and researchers (73, 74). To protect the privacy of workers while gathering personal data, all stakeholders need to take their responsibility for the use of the involved technology (48).

The engineers and researchers have the responsibility to design the technology in such a way that it guarantees the privacy of the user and supports the user in his/her ability to react autonomously (75–77). However, engineers and researchers often do not offer sufficient insight into what constitutes a responsible use of their designs (12). Technologies are never value neutral (69), and it is important that engineers and researchers explore how the development and implementation of their technologies influence and mould not only the ethical values, such as privacy, but also the autonomy of employers and workers and help them reflect on this explorative process (75–77). The responsibility of engineers and/or researchers should focus on perspectives such as value-sensitive design, critical technical practise, reflective design, and values in design (78, 79).

The reflection on the responsibility of workers and employers is not a one-time action. As stated before, differences in interpretations of responsibilities can cause significant problems between workers and employers (30), and the use of technology often alters the original function (26). When using (new) technologies, workers and employers should discuss the responsibilities and intended actions of these technologies with the designers. This discussion should also entail the continuous reflection of the employer to determine whether the conscientious autonomy of the worker has improved. In the case of the healthcare professionals, sensor technologies enabled workers to take responsibility to target work-related health parameters within the workplace. In general, however, these technologies are most effective when workers feel autonomous to self-regulate health-relevant actions (50). Thus, employers should be alert for unintended effects of sensor technologies and ensure an environment that facilitates workers to take their responsibility. When workers and employers share values, such as health, technologies that support the personal goals of workers could increase a sense of conscientious autonomy, thereby improving the self-regulation of healthy behaviour.

### Limitations

The context-specific approach of doing ethics has been a valuable method to investigate the core ethical principles of the digital health technologies in the case studies. In specific this approach helped to obtain a contextual conceptualisation of the ethical principles in the design and implementation of these technologies. However, we realised that this approach was not utilised to its full extent.

Multiple useful tools are now available to help researchers perform responsible research and innovation (e.g., rri-tools.eu). However, at the start of the project SPRINT@Work, approaches to incorporate an ethics structure throughout the complete research cycle of multidisciplinary projects were lacking or at least not commonly practised. Therefore, we started our journey with no clear approach in mind, and we developed our knowledge and approach as we went on.

In the case of the health care workers, this resulted in little attention to the predefined norms and values of the activity tracker. If we would have identified these norms and values before the start of the field studies, the researchers could have incorporated them in the design of the study. This could have prevented negative experiences of workers with the activity tracker.

In the case of the firefighters, we did not involve a specialist in the field of privacy law to help find potential legal solutions for data sharing while protecting the privacy of the workers. The project team would have benefitted from actively involving a privacy specialist from the start of the project. This could have influenced the approach taken by the researcher that designed and implemented the technology, the technology itself, and its suitability for future use.

At last, it can be stated that the problem of compartmentalisation applies to us researchers as well. In order to be able to manage this extensive project, the individual research projects of the PhD candidates were defined as much as possible. Although this approach was meant to save time, it resulted in delays both in the individual research projects concerning the development as in the projects concerning the implementation of the new technologies.

Based on these limitations, we advise multidisciplinary teams to start exploring potential critical ethical issues right from the start of the project. They could use this paper as a first lead on how such issues could be identified. Even though it might not be their initial field of expertise, we appeal to future multidisciplinary teams to also report their findings and possible adjustments to our proposed approach.

## Conclusion: Call for an Overdue Discussion

Based on a substantial literature review, we aimed to discuss the importance of context-specific ethics in design and implementation of digital health technologies. Focusing on a contextual conceptualisation of the core ethical principles in the design and implementation of digital health technologies helps to avoid compartmentalisation, out-of-context generalisation, and neglect of identifying responsibilities. Although it is a long reiterative process in which all stakeholders need to be included in order to assess all critical ethical issues sufficiently, this process is crucial to achieving the intended goal of a technology. We call for multidisciplinary teams, including relevant stakeholders, involved in innovation practises in workplace health promotion to publish their way of doing ethics. Future research teams can learn from these experiences and use and improve their approaches.

Finally, having laid out the landscape and problems of ethics surrounding technologies for workplace health promotion, we believe that an evaluation of policies and standards and a very overdue discussion guided by the signalled ethical problems are needed. Laws and regulations aim to offer protection to users of new technologies, but tend to focus on data access and privacy. Thereby, they leave out other values, such as responsibility and autonomy, which are in close interplay with privacy. It is, therefore, important that engineers and researchers of workplace health promotion themselves enter this debate. They should consider how the design and implementation of their technologies influence and mould the values of the users and adapt their technologies to protect the user from harm, and increase the acceptance. However, it does not stop there. They should also enter the debate about how policies and standards hinder or promote workplace health promotion.

## Data Availability Statement

The data analysed in this study is subject to the following licenses/restrictions: The datasets generated for the case studies that were analysed in this article are available on request to the corresponding author. Requests to access these datasets should be directed to Els Lisette Maria Maeckelberghe, e.l.m.maeckelberghe@umcg.nl.

## Ethics Statement

The studies involving human participants were reviewed and approved by Medical Ethical Exam Committee of the University Medical Center Groningen. The patients/participants provided their written informed consent to participate in this study. Written informed consent was obtained from the individual(s) for the publication of any potentially identifiable images or data included in this article.

## Author Contributions

CR, MJ, AB, and EM contributed to the conception and design of this study and contributed to the data analysis. CR, MJ, and AB contributed to the design of the case studies and data acquisition. All authors contributed to the article and approved the submitted version.

## Conflict of Interest

The authors declare that the research was conducted in the absence of any commercial or financial relationships that could be construed as a potential conflict of interest.

## Publisher's Note

All claims expressed in this article are solely those of the authors and do not necessarily represent those of their affiliated organizations, or those of the publisher, the editors and the reviewers. Any product that may be evaluated in this article, or claim that may be made by its manufacturer, is not guaranteed or endorsed by the publisher.
